# Advances in the study of the relationship between *Porphyromonas gingivalis* and various diseases

**DOI:** 10.3389/fcell.2025.1480233

**Published:** 2025-08-06

**Authors:** Qinghua Zou, Hua Xie, Wentai Yang, Jiacheng Xu, Shuyan Ying, Xiaomin Liao, Jun Xie, Xiongjian Wu, Fan Meng

**Affiliations:** ^1^ The First Clinical College of Medicine, Gannan Medical University, Ganzhou, Jiangxi, China; ^2^ Department of Gastroenterology, The First Affiliated Hospital of Gannan Medical University, Ganzhou, Jiangxi, China; ^3^ The First Affiliated Hospital of Jiangxi Medical College, Nanchang University, Nanchang, Jiangxi, China

**Keywords:** *Porphyromonas gingivalis*, oral cancer, colorectal cancer, oesophageal cancer, pancreatic cancer, periodontal disease

## Abstract

The relationship between the imbalance of flora and the development of various diseases is one of the hotspots of research in recent years. A number of studies have confirmed that *porphyromonas gingivalis* (*P. gingivalis*) is strongly associated with the progression of periodontal disease, oral cancer, esophageal carcinoma, colorectal carcinoma, pancreatic cancer, Alzheimer’s disease, rheumatoid arthritis and other diseases. These diseases have a great impact on human health. Therefore, exploring the pathogenic mechanisms of *P. gingivalis* and the aforementioned diseases is of great significance. In this paper, we focus on the pathogenicity factors of *P. gingivalis* and the relationship between *P. gingivalis* and the progression of various diseases through different signaling pathways, so as to understand the pathogenic mechanism of *P. gingivalis* in a more in-depth and systematic manner.

## 1 Introduction

The relationship between the imbalance of flora and the development of various diseases is hot research topics at the moment (Gao et al., 2018; Cao et al., 2024). Relevant studies have shown that poor oral hygiene can lead to flora imbalance and increase the risk of periodontal disease (Hajishengallis et al., 2012), oral cancer (Chen et al., 2021), esophageal carcinoma (Chen et al., 2015), colorectal carcinoma (Cheng et al., 2020), pancreatic cancer (Pourali et al., 2024), Alzheimer’s disease (Zhang Y. et al., 2024), rheumatoid arthritis (Li Y. et al., 2022) and other diseases. The imbalance of flora is often closely related to factors such as smoking (Apatzidou, 2022), sugar intake (Ge et al., 2021), and antimicrobial use (Rashid et al., 2012). A team of researchers from the Chinese University of Hong Kong recently wrote in *Cell*: in addition to *Helicobacter pylori* (*H. pylori*), *Streptococcus anginosus* (*S. anginosus*) is also a pathogenic bacterium that promotes gastric cancer (Fu et al., 2024). The virulent surface protein TMPC binds to annexin A2 (ANXA2) on gastric epithelial cells, triggering downstream activation of bacterial attachment, invasion, and carcinogenic mitogen-activated protein kinase (MAPK) signaling. This clearly demonstrates that dysbiosis influences the onset and progression of disease, and that microbes may also act synergistically to collectively promote the development of certain disorders.

There are more than 700 kinds of oral bacteria, of which *Treponema denticola*, *porphyromonas gingivalis* (*P. gingivalis*) and *Tannerella forsythia* are called “red complex” (Aas et al., 2005). *P. gingivalis*, which is low in abundance but has a strong influence on the oral microflora, is known as a “keystone species” (Darveau et al., 2012). Epidemiological studies indicate that *P. gingivalis* is detected in 50% to 80% of patients with severe periodontitis, significantly higher than in healthy controls (10%–30%). The highest infection rates are observed in Asian populations (particularly China and Japan), while lower detection rates are reported in European and American countries. Notably, *P. gingivalis* colonization is detectable in 37% of individuals aged 0–18 years, suggesting potential early microbial establishment. We have previously confirmed that *P. gingivalis* is a specific pathogen that promotes the progression of esophageal squamous cell carcinoma (Meng et al., 2019). It can activate the nuclear factor-κB (NF-κB, dysfunction of it can lead to inflammatory related diseases and cancer) pathway to promote the proliferation and migration of esophageal squamous cell carcinoma. *P. gingivalis* is a non-glycolytic, gram-negative anaerobic bacterium that produces outer membrane vesicles (OMVs) (Fan et al., 2023), lipopolysaccharide (LPS) (Stasiewicz and Karpiński, 2022; Li et al., 2021), gingipains (Travis et al., 1997) and other virulence factors. These virulence factors can help *P. gingivalis* better invade host cells and cause damage to body tissues. They can activate the immune system through multiple mechanisms and promote the production of inflammatory mediators, with implications for cancer and other diseases. This review primarily provides a detailed exploration of the molecular pathways linking the virulence factors of *P. gingivalis* to disease pathogenesis. It aims to offer insights for both basic researchers and clinical scientists, with the hope of proposing new research directions at the intersection of microbiology, immunology, and clinical medicine. Here, we introduce some pathogenic factors of *P. gingivalis in detail*:

The outer membrane vesicles are bilayer spherical membrane structures with a diameter of 50–250 nm, containing proteins, lipids, nucleic acids, and other biologically functional molecules (Zhang Z. et al., 2020). Their production is regulated by factors such as the expression of the fimA gene, autolysins, gingipains, and PPAD (a unique peptidyl deiminase) (Vermilyea et al., 2021). The process of formation may be as follows: The accumulation of misfolded or overexpressed envelope proteins increases the pressure of the outer membrane, and “budding” is formed at the site where the connection between the outer membrane and peptidoglycan is missing. The “budding” of vesicles leads to increased local curvature of the bacterial outer membrane (Bohuszewicz et al., 2016). The outer membrane vesicles are deployed by *P. gingivalis* to selectively coat and activate neutrophils,and they trigger degranulation without affecting neutrophil viability. Granular ingredients with antibacterial activity are degraded by powerful proteases bound by OMVs to ensure the survival of bacteria (du Teil Espina et al., 2022). Small RNA molecules in OMVs have potential gene regulatory functions as interspecific communication molecules. Some studies have suggested that sRNA45033 packaged by *P. gingivalis* OMVs can inhibit the expression of CBX5 (Chromobox 5, also known as heterochromatin protein 1 alpha, is a structural protein) in host cells and reduce the H3K9me3 (trimethylated of lysine 9 of histone H3) level of p53, resulting in increasing the expression of p53 and promoting host cell apoptosis (Fan et al., 2023).

LPS consists of three elements: O antigen, core polysaccharide,and lipid A (Qin et al., 2024). The key structure of LPS is the phosphorylated glucosamine disaccharide and the fatty acid lipid A,which can be specifically and sensitively recognized by the innate immune system (Herath et al., 2021). *P. gingivalis* has multiple forms of lipid A, and the lipid A domain influences the biological activity of host cells. Changes in domain fatty acids, the level of phosphorylation, and the deletion of phosphate or monosaccharide groups will affect the biological activity of host cells. LPS can promote cell proliferation and produce interleukin-1β (IL-1β), IL-6, and IL-8 (Kato et al., 2014). LPS is recognized by TLR4 (Toll-like receptor 4, TLR (Ferwerda et al., 2008) is a transmembrane glycoprotein that is expressed on the plasma membrane or the inner membrane of the cell,and it can form homologous or heterodimers and undergo conformational changes when ligands bind). TLR4 stimulates the myeloid differentiation primary response factor 88 (MyD88) to release nuclear factor κB (Stasiewicz and Karpiński, 2022).

Cysteine proteases from *P. gingivalis* have trypsin-like activity, which are important virulence factors in periodontitis, and they are called gingipains (Travis et al., 1997). Gingipains are classified into two primary categories: arginine-specific gingival proteases (Rgps, which can be subdivided into RgpA and RgpB) and lysine-specific gingival proteases (Kgps) (Nonaka et al., 2022). Gingipains play a role in host colonization, defense inactivation, iron and nutrient acquisition, and tissue destruction (Dominy et al., 2019). Firstly, Rgps are capable of binding to a variety of extracellular matrix proteins, thereby enhancing their adhesion within the host. Secondly, gingipains can proteolytically inactivate cationic antimicrobial peptides. They can degrade cytokines, complement formation, and several receptors, which collectively undermine host defense mechanisms (Imamura et al., 2003). Furthermore, gingipains degrade hemoglobin to liberate heme, facilitating the acquisition of nutrient peptides through a protein hydrolysis system composed of gingival protease-containing endopeptidases, oligopeptidases, and dipeptidyl and tripeptidyl peptidases, operating in a cascading manner. Ultimately, gingipains disrupt the equilibrium of proteolysis between host proteases and endogenous protease inhibitors, contributing to tissue destruction.

The fimbriae of *P. gingivalis* are filamentous surface appendages that extend from the outer membrane, consisting of two forms: long fimbriae composed of the FimA subunit and short fimbriae containing the Mfa1 subunit protein (Hasegawa and Nagano, 2021). Fimbriae enhance biomembrane formation, bacterial motility, bacterial adhesion to host cells, and bacterial invasion of cells (Xu et al., 2020). Each type of fimbriae consists of five FimA (A-E) and five Mfa1 (1–5) proteins, which are regulated by the fim and mfa gene clusters, respectively (Hasegawa and Nagano, 2021). Long fimbriae can activate NF-κB by binding TLR2, which promotes the production of pro-inflammatory cytokines, including IL-6, IL-1β, IL-8, and tumor necrosis factor-α (TNF-α) (Wielento et al., 2022). Long fimbriae also disrupt immune clearance by hijacking the complement pathway (Hajishengallis et al., 2008).

## 2 Periodontal disease

Periodontal disease is a chronic oral disease, including periodontitis and gingivitis, which can lead to bleeding and swelling of the gums, tooth loss, and its prevalence has increased year by year (Janakiram and Dye, 2020). There are many causative factors for periodontal disease, including dental plaque, tartar, tooth surface staining, food impaction, trauma, smoking, age, heredity, diabetes, sex hormones, psychological factors, of which dental plaque is the main cause. *P. gingivalis* has a great influence on the development of periodontitis, which colonizes the subgingival area and triggers periodontitis, producing virulence factors that further damage periodontal tissues (Hajishengallis et al., 2012).

**FIGURE 1:**
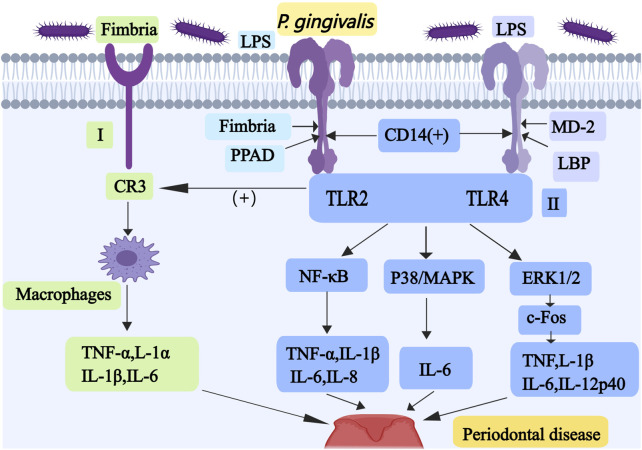
Mechanisms associated with periodontal disease caused by *P. gingivalis* I, Fimbriae-induced TLR2 promotes the interaction between *P. gingivali*s and CR3; it stimulates the production of TNF-α, IL-1α, IL-1β, and IL-6 in macrophages. II, The LPS of *P. gingivali*s may activate NF-κB, p38/MAPK and ERK1/2 pathways through TLR2 or TLR4 to promote the production of inflammatory substances. Abbreviations: TLR, Toll-like receptor; CR3, complement receptor 3; NF-κB, nuclear factor κB; MAPK, mitogen activated protein kinase; ERK, extracellular signaling kinase.

The outer membrane vesicles have strong inflammatory properties, and they can regulate neutrophils and macrophages, facilitating the invasion of oral epithelial cells (Ma and Cao, 2021). *P. gingivali*s can cause immune cells to release many pro-inflammatory factors and enhance its inflammatory damage and immune evasion by promoting the proliferation of immunosuppressive cells (Su et al., 2017). When proinflammatory cytokines (such as TNF-α, IL-1, IL-6, IL-11,and IL-17) reach critical concentrations, periodontal tissue damage occurs. *P. gingivali*s can induce mitochondrial dysfunction by altering mitochondrial metabolic status, quality control, production of reactive oxygen species (ROS) and the mediation of apoptosis, which promote the occurrence of periodontitis (Luo et al., 2024). After infection by *P. gingivali*s, the gingival fibroblasts were transformed to pro-inflammatory phenotype. And they secreted proteolytic enzymes and induced osteoclast formation (Wielento et al., 2023). The invasion of *P. gingivali*s into osteoblasts downregulates the expression of transcription factors core-binding factor alpha-1 (Cbfa-1) and Osterix, which inhibits osteoblast differentiation and osteogenesis (Zhang et al., 2010). Osterix is a zinc-finger osteoblast-specific transcription factor, which regulates various genes involved in the differentiation and maturation of bone cells (Liu et al., 2020). The LPS of *P. gingivali*s may activate NF-κB, p38/mitogen activated protein kinase (MAPK), and extracellular signaling kinase (ERK) 1/2 pathways through TLR2 or TLR4, thereby promoting the production of inflammatory mediators (as shown in Figure 1) (Ding et al., 2013). Binding of TLR2 to LPS requires the assistance of fimbriae, CD14, and peptidylarginine deiminase (PPAD), and the absence of any one of these may block its binding (Wielento et al., 2022).

Fimbriae-induced TLR2 also promotes the interaction between *P. gingivali*s and complement receptor 3 (CR3), which activates the ligand-binding capacity of CR3, thereby stimulating the production of TNF-α, IL-1α, IL-1β, and IL-6 in macrophages (as shown in Figure 1) (Wang et al., 2007). TLR4 must form a dimeric complex with myeloid differentiation factor 2 (MD-2) in order to capture its ligand LPS. LPS-binding protein (LBP) and CD14 improve the efficiency of LPS transport and the sensitivity of the assay (Ryu et al., 2017). Proinflammatory cytokines including TNF-α, IL-1β, IL-6, and IL-8, are induced by the activated NF-ĸB pathway. p38/MAPK pathway upregulates IL-6 expression in monocytes (Pollreisz et al., 2010). ERK1/2 mainly activates c-Fos to increase production of the pro-inflammatory cytokines TNF, IL-1β, IL-6, and IL-12p40 (Lucas et al., 2021). Some researchers have produced red cell membrane nanovesicles that can accurately target and adhere to *P. gingivali*s, which can inhibit *P. gingivali*s and prevent its invasion of epithelial cells, thus alleviating the progression of periodontitis (Tang et al., 2024). Some researchers have also produced a targeting nanoagent antibody-conjugated liposomal drug carrier with ginsenoside Rh2 (ALR), which can reduce the proportion of *P. gingivali*s and maintain a relatively stable oral flora (Chen et al., 2023). Studies have shown that *bacteroides* glutaminyl cyclases can inhibit *P. gingivali*s, which is a promising target for drug development in the treatment of periodontitis (Taudte et al., 2021).

In summary, *P. gingivalis* primarily contributes to periodontitis pathogenesis through three key mechanisms: promoting oral inflammatory responses, destroying periodontal tissues, and disrupting the osteoblast-osteoclast homeostasis. This pathogenic process involves multiple virulence factors comprising OMV, LPS, and fimbriae, along with proinflammatory cytokines and key osteogenic transcription factors including Osterix (as shown in Figure 1).

## 3 Oral cancer

Oral cancer is one of the most prevalent malignant cancers in the world, with more than 90% being oral squamous cell carcinoma (OSCC) (Bagan et al., 2010). Factors known to promote OSCC include tobacco, alcohol consumption, betel nut chewing, viral infections, diet, deficiencies in vitamins and minerals, occupational exposures, and hereditary diseases. However, a growing number of epidemiologic, clinicopathologic, and molecular studies have demonstrated that oral microorganisms, such as *P. gingivali*s, also have an effect on the carcinogenesis of OSCC (Chen et al., 2021). *P. gingivali*s may contribute to oral carcinogenesis mainly through the following mechanisms:

### 3.1 *P. gingivalis* induces epithelial-mesenchymal transition (EMT) to promote oral cancer

**FIGURE 2:**
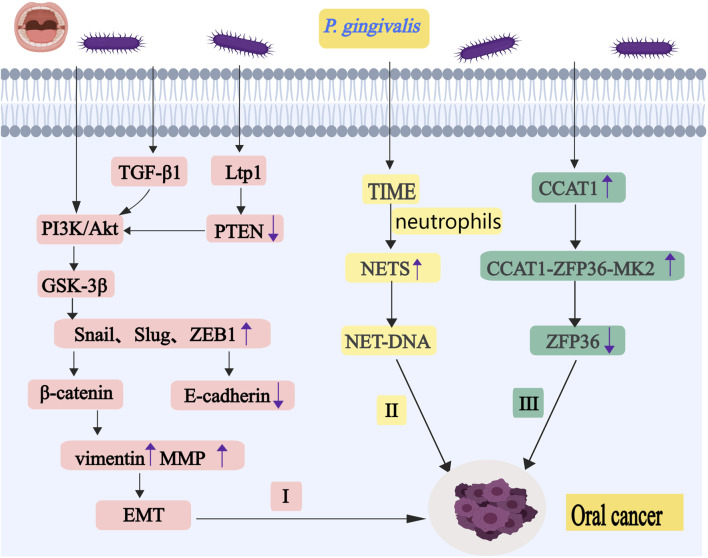
*P. gingivalis* promotes the occurrence and development of oral cancer by regulating EMT, ZFP36 and NETs. I, *P. gingivali*s increases activation of the PI3K/Akt pathway. It promotes phosphorylation of GSK-3β, leading to the upregulation of Snail, Slug, and ZEB1. *P. gingivali*s also can induce TGF-β1 and LMWTP to regulate protein kinase Akt. These eventually lead to the occurrence of EMT. II, Neutrophils release NETs under the stimulation of *P. gingivali*s,and it may stimulate cancer cell migration through the chemotactic effect of NET-DNA. III, *P. gingivali*s upregulates CCAT1 expression. It can form a complex with ZFP36 and MK2. This complex ultimately attenuates the inhibitory effects of ZFP36 on key downstream oncogenes. Abbreviations: PI3K, phosphatidylinositol 3-kinase; GSK-3β, glycogen synthase kinase-3β; TGF-β1,t ransforming growth factor-β1; LMWTP, low molecular weight tyrosine phosphatase; EMT, epithelial-mesenchymal transition; NETs, neutrophil extracellular traps; CCAT1, colon cancer-associated transcript-1; MK2, MAPK-activated protein kinase 2.

Treatment of OSCC cells with heat-inactivated *P. gingivali*s or *F. nucleatum* resulted in a decrease in E-cadherin (cell adhesion molecule, a transmembrane glycoprotein recognized as a marker of EMT in epithelial cells) and an increase in vimentin (mesenchymal intermediate filaments, which are not expressed in normal epithelial cells, are associated with an aggressive tumor phenotype). These findings indicate that the cells underwent EMT (Abdulkareem et al., 2018). EMT is manifested as epithelial cells losing their typical epithelial characteristics and transitioning into cells with mesenchymal characteristics. There are three subtypes of EMT, type 1 EMT plays a role in embryogenesis and organ development. It does not cause fibrosis and does not induce an aggressive phenotype. Type 2 EMT plays a role during organ fibrosis, wound healing and regeneration. It usually occurs after tissue damage. Type 3 is involved in the development of cancer. After undergoing EMT, the cancer cells exhibit the following characteristics: motility, invasiveness, stemness, and increased resistance to drugs (Santos et al., 2019). *P. gingivali*s can invade and colonize the host cell surface with the help of its fimbriae (Jia et al., 2019). EMT can occur in all three of the following ways. First, *P. gingivali*s increases activation of the phosphatidylinositol 3-kinase (PI3K)/Akt (a serine/threonine kinase) pathway, which promotes phosphorylation of glycogen synthase kinase-3β (GSK-3β), leading to the upregulation of Snail, Slug, and ZEB1 (as shown in Figure 2) (Lee et al., 2017). Snail, Slug, and ZEB1 promote the transformation of EMT, resulting in a decrease in E-cadherin and atypical activation of β-catenin. Changes in the subcellular localization of β-catenin increase the expression of the mesenchymal markers vimentin and matrix metalloproteinase (MMP)-2, 7, and 9. Second, *P. gingivali*s induces the extracellular secretion of transforming growth factor-β1 (TGF-β1), which activates the regulator of the protein kinase Akt (as shown in Figure 2) (Hao et al., 2019). Third, *P. gingivali*s secretes a low molecular weight tyrosine phosphatase (LMWTP, also referred to as Ltp1) in epithelial cells, which dephosphorylates PTEN (a phosphatase and tensin homologue deleted from chromosome 10), leading to proteasome degradation and reducing the inhibition of the PI3K/Akt pathway (as shown in Figure 2) (Liu C. et al., 2021). *P. gingivali*s increases oral cancer cell invasiveness by inducing EMT-like changes and expression of the cancer stem cell (CSC) markers CD44 and CD133, while the production of MMP degrades extracellular matrix and basal components, which also promotes cancer invasive metastasis (Ha et al., 2015).

### 3.2 *P. gingivali*s downregulates ZFP36/TTP expression to promote oral cancer progression

ZFP36 (also known as tristetraprolin, TTP) belongs to a family of zinc finger proteins that bind to AU-rich binding sites within the 3′-untranslated regions of specific mRNAs to promote mRNA deadenylation and decay, and it is a regulator of many pro-inflammatory proteins that can inhibit immune responses (Cook et al., 2022). Its reduced expression will promote the progress of the tumor (Lee et al., 2022). Persistent infection of oral epithelial cells by *P. gingivali*s downregulates ZFP36 protein expression and upregulates CCAT1 (colon cancer-associated transcript-1, also known as cancer-associated regional long-stranded non-coding RNA-5) expression, which enhances cancer-associated biological behaviors in human oral epithelial cells (as shown in Figure 2) (Lu et al., 2024). CCAT1, when highly expressed, can form a complex with ZFP36 and MK2 (MAPK-activated protein kinase 2, recognized as the major protein controlling ZFP36), which acts as a molecular scaffold and leads to an increase in the binding affinity between MK2 and ZFP36. This complex ultimately inhibits ZFP36 phosphorylation and attenuates the inhibitory effects of ZFP36 on key downstream oncogenes.

### 3.3 *P. gingivalis* promotes oral cancer progression by inducing neutrophils to release NETs

Neutrophils release neutrophil extracellular traps (NETs) under the stimulation of inflammatory mediators and microorganisms, which consist of extracellular chromatin decorated by histones and many granule proteins (Mutua and Gershwin, 2021). In recent years, NETs have been shown to play an important role in multiple aspects of tumorigenesis and progression, including promotion of tumor growth, proliferation, and enhancing immune evasion (Kaltenmeier et al., 2021). Some studies have suggested that the structure of NETs can be destroyed by DNase I, but the protein components of NETs cannot be fully degraded (Prince et al., 1998). *P. gingivalis* can stimulate the release of NETs in the tumor immune microenvironment (TIME), which leads to the progression of OSCC (as shown in Figure 2) (Guo et al., 2024). Some studies have suggested that NET release of NE activates the TLR4-p38-PGC-1α axis in cancer cells to increase mitochondrial biogenesis (Yazdani et al., 2019). Peroxisomes proliferator-activated receptor gamma coactivator 1-alpha (PGC-1α) can affect mitochondrial respiration, detoxification of ROS, fatty acid oxidation (FAO), and glucose-or-glutamine-derived fat production in cancer cells (Fakhri et al., 2024). NETs can also maintain mitochondrial homeostasis by affecting fission, fusion, and autophagy. NETs may stimulate cancer cell migration through the chemotactic effect of NET-DNA (a chemokine that attracts cancer cells) on DNA sensors, such as CCDC25 (a transmembrane protein), and the targeting of CCDC25 may be an attractive therapeutic strategy to prevent metastasis (Yang L. et al., 2020). CCDC25 is a specific sensor for DNA, specifically 8-OHdG-rich DNA present in NETs. After sensing NET-DNA to the extracellular domain amino acids 21–25, CCDC25 recruits integrin-linked kinase through its intracellular C-terminal and activates β-parvin (an adaptor protein that binds to the integrin-linked kinase) -RAC1 (which is involved in tumorigenesis, proliferation, metastasis events and development of drug resistance (De et al., 2019)) -CDC42 (cell division control protein 42) cascade to induce cytoskeletal rearrangement and targeted migration of tumor cells. NETs can also activate NF-κB signaling to promote not only the proliferation, migration, and invasion of breast cancer cells (Zhu et al., 2021), but also the metastasis of non-small cell lung cancer (Wang Y. et al., 2022). Overall, NETs are involved in the progression of various cancers.

In the development and progression of oral cancer, *P. gingivalis* promotes EMT by modulating Snail, Slug, and ZEB1, leading to downregulation of E-cadherin and upregulation of vimentin and MMP. Infection of oral epithelial cells by *P. gingivalis* downregulates the expression of ZFP36 protein, which promotes oral carcinogenesis. Additionally, *P. gingivalis* influences the tumor immune microenvironment by inducing neutrophil extracellular traps (NETs), which stimulate cancer cell migration through NET-DNA-mediated chemotaxis of DNA sensors such as CCDC25 (as shown in Figure 2).

## 4 Esophageal cancer

Esophageal carcinoma, primarily divided into esophageal squamous cell carcinoma (ESCC) and esophageal adenocarcinoma (EAC), is more common in developing countries (Abnet et al., 2018). ESCC patients have a significantly lower diversity of oral microbiota than healthy individuals and those with atypical hyperplasia (Chen et al., 2015). Using 16S rRNA sequencing, the enrichment of *P. gingivali*s in the oral microbiome is much higher than in healthy individuals. This article focuses on the following mechanisms through which *P. gingivalis* affects ESCC.

### 4.1 *P. gingivalis* reduces *PDCD4* activity to promote ESCC progression

**FIGURE 3:**
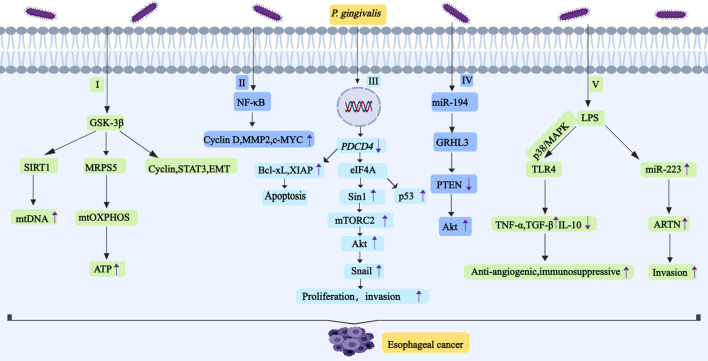
*P. gingivalis* promotes the development and progression of esophageal cancer by regulating GSK-3β, NF-κB, *PDCD4*, miR-194/GRHL3/PTEN/Akt,TLR4 and ARTN. I, *P. gingivali*s induces high expression of GSK-3β, SIRT1 and MRPS5 in ESCC. Activation of SIRT1 enhances mtDNA replication. Deacetylated MRPS5 in mitochondria can induce mtOXPHOS to produce ATP. Inhibition of GSK-3β downregulates the expression of cyclin. GSK-3β can regulate the activity of STAT3,and it also induces the occurrence of EMT. II, *P. gingivali*s activates NF-κB signal pathway and then upregulates the expression of cyclin D1, MMP2 and c-MYC. III, *P. gingivalis* reduces the activity of *PDCD4*, this will increase the activity of eIF4A.And eIF4A promotes Sin1 translation, which leads to the increase of mTORC2 activity, thus activating Akt to promote Snail expression. IV, *P. gingivali*s may promote esophageal cancer proliferation and migration through miR-194/GRHL3/PTEN/Akt signaling axis,it leads to downregulation of PTEN levels and upregulation of p-Akt levels. V, LPS-TLR4 signaling is related to the activation of ERK and p38/MAPK signaling pathways. TLR4 connection increases the expression of TNF-α and TGF-β, and it decreases the expression of IL-10. LPS promotes the expression of miR-223, and miR-223 promotes the expression of ARTN. Abbreviations: GSK-3β, glycogen synthase kinase-3β; SIRT1, silent information regulator 1; MRPS5, mitochondrial ribosomal protein S5; STAT3, transcription activator 3; EMT, epithelial-mesenchymal transition; *PDCD4*, programmed cell death Factor 4; eIF4A, eukaryotic initiation factor 4A; ARTN, artemisinin.

Programmed cell death factor 4 (*PDCD4*) is a tumor suppressor gene, which can inhibit cell growth, tumor invasion and metastasis, and it induces cell apoptosis. *PDCD4* is widely expressed in tissues, and the protein is located in either the cell nucleus or the cytoplasm (Matsuhashi et al., 2019). Eukaryotic initiation factor 4A (eIF4A) is an ATP-dependent RNA helicase. The binding of PDCD4 to it inhibits its activity, resulting in the translation of mRNA containing structured 5′-UTR being blocked (as shown in Figure 3) (Wedeken et al., 2011). The depressant effect of *PDCD4* on the translation of p53 mRNAs is mediated by the 5′-UTRs of the p53 mRNAs, leading to the inhibition of p53 expression, but the expression of p53, in turn, reduces the level of *PDCD4* protein (Yang et al., 2023). *PDCD4* can affect the luciferase translation of SIN1 5′-UTR fusion, which is concentration-dependent. Stress-activated protein kinase interacting protein 1 (Sin1) is the mammalian target of rapamycin complex 2 (mTORC2, which mediates the metabolism of cell growth and promotes the occurrence of tumors). Inhibition of Sin1 translation leads to inhibition of mTORC2 activity, which in turn inhibits Akt activation and Snail expression, ultimately weakening tumor proliferation and invasion (as shown in Figure 3) (Wang et al., 2017). *P. gingivalis* reduces the activity of *PDCD4*, which enhances cancer cell stemness and leads to CSC enrichment in ESCC cells (Li et al., 2023). *PDCD4* blocks the formation of translation-initiation complexes by binding to internal ribosomes, which reduces translation of the anti-apoptotic proteins Bcl-xL and XIAP. Bcl-xL (B-cell lymphoma-extra-large) is one of the homologs of the B-cell lymphoma protein family (the anti-apoptotic Bcl-2), which can promote tumor stem cell properties. XIAP (X-linked IAP, which belongs to the IAP family of apoptosis protein inhibitors) is the only member of the family that inhibits apoptosis by directly binding to and inhibiting cysteine asparaginase.

### 4.2 *P. gingivali*s induces high GSK-3β expression to promote ESCC progression

GSK-3β is a multifunctional serine/threonine kinase whose aberrant activation promotes malignant proliferation and distant metastasis in many types of tumors (Lin et al., 2020). The expression of GSK-3β in ESCC tissues is high, and its expression is proportional to the infection time of *P. gingivali*s, which indicates that *P. gingivali*s induces the high expression of GSK-3β in ESCC cells in a time-dependent manner (Liu et al., 2023). *P. gingivali*s induces high expression of GSK-3β, silent information regulator 1 (SIRT1) and mitochondrial ribosomal protein S5 (MRPS5) in ESCC, and promotes oxidative phosphorylation of mitochondrial oxidative phosphorylation (mtOXPHOS) to produce large amounts of ATP (as shown in Figure 3). SIRT1 is an enzyme that can catalyze the deacetylation of a variety of proteins, and it plays a crucial role in cell cycle, DNA repair, oxidative stress, and metabolic remodeling (Singh and Ubaid, 2020). Activation of SIRT1 enhances mtDNA replication (Sun et al., 2020). MRPS5 is a member of the 28S small subunit of the mitochondrial ribosomal protein, which participates in mitochondrial biosynthesis. Deacetylated MRPS5 in mitochondria can induce mtOXPHOS to produce ATP, which has a certain influence on the malignant process of tumors (Akbergenov et al., 2018). Inhibition of GSK-3β causes ESCC cells to stall in G0/G1 and G2/M phases, and it also downregulates the expression of cyclin D1 and cyclin dependent kinase 4 (CDK4) and upregulates the expression of cyclin B1 (Bolidong et al., 2020). While cyclin D1 expression is tightly controlled in normal cells, it becomes overexpressed in cancers. CDK4 and CDK6 bind to cyclin D, which drives the cell transition from the G1 phase of the cell cycle to the S phase when DNA is synthesized (Goel et al., 2022). Cyclin B1 can play a role in the transition from G2 phase to M phase of the cell cycle. GSK-3β promotes the development of ESCC by altering the activity of signal transducer and activator of transcription 3 (STAT3) (Gao et al., 2017). As described earlier in this article, GSK-3β also induces the occurrence of EMT and promotes tumor progression (Liu et al., 2018).

### 4.3 *P. gingivali*s mediates the expression of B7-H4 and KDM5B to promote the progress of ESCC

B7-H4 (It is also called B7S1 or B7x) belongs to the B7 superfamily, which is widely expressed in many types of cancers and interacts with the B7-H4 receptor. Activated T cells produce B7-H4 receptors that transmit inhibitory signals to downregulate T cell function. Interaction of B7-H4 with B7-H4 receptors can inhibit tumor immunity (Miao and Sun, 2021). B7-H4 activates the signal transducer and STAT3 (It can activate cytokines and growth factors, which promotes the transmission of signals from the cell surface to the cell) pathway that promotes the secretion of IL-6, and IL-6 promotes the expression of B7-H4 through binding to the IL-6 receptor, ultimately promoting the development of esophageal squamous cell carcinoma (Chen et al., 2016). Overexpression of B7-H4 is associated with malignancy and poor prognosis in CRC, and B7-H4 expression can lead to downregulation of E-cadherin expression and upregulation of vimentin during EMT (Yan et al., 2022). Elevated levels of lysine demethylase 5B (KDM5B) have been found in a variety of human cancers, and it is considered to be a transcriptional suppressor associated with tumor growth, angiogenesis, invasion, metastasis, and tumor-related chemotherapy resistance (Zheng et al., 2019). KDM5B has a wide range of regulatory roles in chromatin structure and represses the transcriptional function of genes, acting as an oncogene (Fu et al., 2020). KDM5B and Transcription factor AP-2 gamma (AP-2γ, it regulates cell proliferation, cell cycle, and apoptosis, and participates in the occurrence of a variety of cancers (Xing et al., 2022)) cooperate with Myc (overexpression of which can lead to tumorigenesis) to inhibit the cell cycle inhibitor p21, and the ectopic expression of KDM5B can also promote the EMT of cancer cells (Cui et al., 2017). *P. gingivalis* promotes esophageal squamous cell carcinoma progression by mediating immune checkpoints B7-H4 and KDM5B (Yuan et al., 2019).

### 4.4 *P. gingivali*s promotes ESCC through other pathways

*P. gingivali*s may also have the following mechanisms to promote the proliferation, migration and invasion of ESCC cells: first. LPS increases the adhesion properties of esophageal cancer cells by TLR4 signaling and selectin ligands (as shown in Figure 3) (Rousseau et al., 2013). In ESCC cells, TLR4 connection increases the expression of TNF-α and TGF-β, while IL-10 expression decreases. TGF-β may inhibit tumor-specific T cell immunity to promote cancer growth. IL-10 is a multifunctional cytokine that can inhibit both angiogenesis and immunity. LPS-TLR4 signaling is related to the activation of ERK and p38/MAPK signaling pathways (Zu et al., 2017). LPS may also increase the expression of artemisinin (ARTN) to affect the biological behavior of ESCC cells (Kong et al., 2023) (as shown in Figure 3). ARTN is a target gene of miR-223, and the expression of miR-223 affects ARTN expression, cell migration and invasion in esophageal cancer cells (Li et al., 2011). Second, as described in our previous article, *P. gingivali*s has an effect on the expression of key molecules in the NF-κB signaling pathway, such as the cell cycle protein D1, MMP2, and c-MYC (Meng et al., 2019) (as shown in Figure 3). MMP can promote extracellular matrix renewal and cancer cell migration, and it also controls cell growth and inflammation (Kessenbrock et al., 2010). Third, *P. gingivali*s may promote esophageal cancer proliferation and migration through miR-194/GRHL3/PTEN/Akt signaling axis (as shown in Figure 3). MiR-194 regulates GRHL3 (a gene-encoded transcription factor that suppresses cancer) and GRHL3 regulates PTEN, which ultimately leads to downregulation of PTEN levels and upregulation of p-Akt levels (Liang et al., 2020). Fourth, the gingipains secreted by *P. gingivali*s induce the expression of pro-inflammatory mediators, such as matrix metalloproteinases, which degrade the extracellular matrix (ECM) and destroy immunoglobulins and complement components C3 and C5 (Malinowski et al., 2019). They make ESCC escape the killing of macrophages and neutrophils and promote the occurrence and development of ESCC.

In esophageal cancer, *P. gingivali*s promotes carcinogenesis by reducing *PDCD4* activity and enhancing cancer stemness. *PDCD4* inhibits the helicase activity of eIF4A through binding, thereby suppressing mRNA translation; it can also directly bind to mRNA to block translation. *P. gingivali*s induces GSK-3β overexpression in ESCC cells in a time-dependent manner. Elevated GSK-3β not only enhances the expression of SIRT1 and MRPS5, leading to increased ATP production, but also influences cell cycle regulators, STAT3 signaling, and EMT (as shown in Figure 3). Furthermore, *P. gingivali*s facilitates tumor progression by regulating immune checkpoint molecules B7-H4 and KDM5B.

## 5 Colorectal carcinoma

Colorectal carcinoma (CRC) is one of the three most common cancers worldwide, and its morbidity and mortality rates remain high (Mármol et al., 2017). With changes in healthier lifestyle choices, the increase in colonoscopy screening and the improvement of treatment, the incidence rate may show a downward trend. Risk factors for CRC generally include a history of precancerous adenomatous polyps, having CRC or precancerous adenomatous polyps in immediate family members, family history of hereditary CRC, inflammatory bowel disease, and poor dietary habits (such as long-term drinking, excessive intake of barbequed, salted, smoked, or high-fat foods). At present, many studies have shown that intestinal flora imbalance is associated with CRC (Cheng et al., 2020).

### 5.1 *P. gingivali*s promotes CRC progression by fermenting dietary fiber to produce butyrate

**FIGURE 4:**
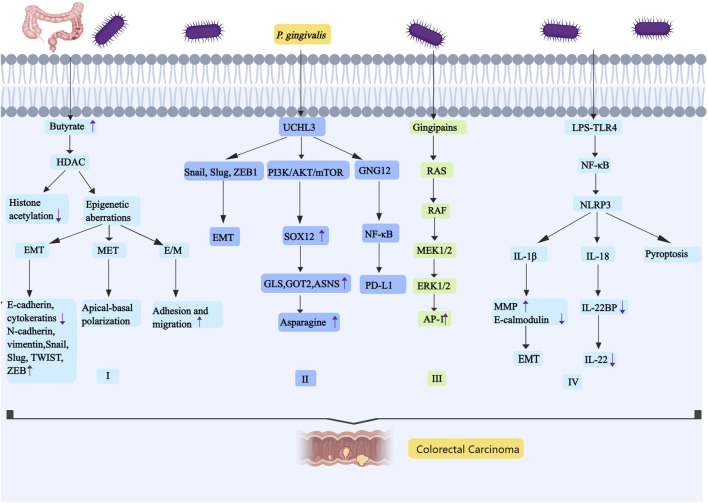
*P. gingivali*s promotes the occurrence and development of colorectal cancer by regulating butyrate, UCHL3, *RAS-RAF*-MEK-ERK and NLRP3. I, *P. gingivalis* has the ability to promote the development of colorectal tumors by secreting butyrate. The carcinogenic mechanism of butyrate may be related to HDAC,and the increase of HDAC expression will decrease the acetylation of histone. The epigenetic aberration caused by the increase of HDAC activity includes three states: EMT, MET and E/M. II, *P. gingivalis* can significantly increase UCHL3. UCHL3 promotes EMT through the upregulation of Snail, Slug, and ZEB1. UCHL3 activates the PI3K/AKT/mTOR pathway, it induces the upregulation of SOX12. SOX12 can promote the synthesis of asparagine by upregulating GLS, GOT2, and ASNS. UCHL3 may also interact with GNG12, and GNG12 promotes tumor progression by up-regulating PD-L1 through activation of the NF-κB pathway. III, *P. gingivalis* can stimulate *RAS-RAF*-MEK-ERK signal pathway, it increases the expression of AP-1. IV, *P. gingivalis* promotes colorectal cancer by activating NLRP3 inflammasome. LPS binds with TLR4 to activate NF- κB, and then NF- κB promotes the expression of NLRP3. The NLRP3 inflammasome induces three caspase-1-mediated reactions: IL-1β, IL-18, and pyroptosis. IL-1β is involved in EMT,and IL-18 downregulates the soluble IL-22 receptor. Abbreviations: HDAC, histone deacetylase; UCHL3, ubiquitin carboxy-terminal hydrolase L3; SOX12, sex-determining region Y -box12; GLS, glutaminase; GOT2, glutamic-oxaloacetic transaminase 2; ASNS, asparagine synthetase; GNG12, G protein subunit gamma 12; AP-1,activating protein 1.

*P. gingivalis* has the ability to promote the development of colorectal tumors by secreting butyrate, and the incidence of colorectal tumors was significantly increased in mice infected with this bacterium, which did not occur when the bacterial butyrate synthesis gene was disrupted (Okumura et al., 2021). Colonic microorganisms ferment dietary fiber to produce butyrate, the preferred energy source for colonic mucosal cells, which can promote the apoptosis of CRC cells (Fung et al., 2011). The discrepancy in the effect of butyrate on tumorigenesis is known as the butyrate paradox (Bultman and Jobin, 2014). The mechanism of butyrate’s pro-tumorigenic effect may be related to histone deacetylase (HDAC), an enzyme whose increased expression results in a decrease in the amount of histone acetylation (as shown in Figure 4). Moreover, histone is a major component of chromatin. When it is acetylated, it regulates gene transcription and inhibits carcinogenesis. Histone acetylation and formylation play important roles in tumor progression, and histone deacetylase inhibitors (HDIs) have emerged as promising anticancer drugs (Damaskos et al., 2017). Epigenetic aberrations caused by increased HDAC activity affect the occurrence and progression of cancer (as shown in Figure 4) (Bayat et al., 2018). EMT is caused by a series of epigenetic changes, including chromatin remodeling and histone modifications. EMT causes loss of connective structures, and adhesion between the parietal and basal polarized epithelial cells disappears. The newly formed mesenchymal cells have high migration ability and aggressiveness (Lamouille et al., 2014). First, throughout the EMT (epithelial-mesenchymal transition) process, cancer cells often undergo molecular events, e.g., decreased levels of epithelial markers (E-cadherin, cytokeratins), decreased levels of mesenchymal markers (N-cadherin, vimentin), and they acquire a high migratory and invasive capacity through the increase in EMT-related transcription factors (TFs) (Snail, Slug, TWIST, ZEB). Second, during mesenchymal-epithelial transition (MET), cells regain epithelial properties. The interepithelial junctions cause acro-basal polarization. Third, the intermediate stage between the whole epithelium and the whole mesenchymal is called the E/M mixed state. Cancer cells in mixed E/M state have both intercellular adhesion and the ability to migrate (Wawruszak et al., 2019). The basic structural classes of HDI include trichostatin A (TSA), valproic acid (VPA). TSA induced EMT reversal by increasing E-cadherin and decreasing vimentin expression. TSA decreased Slug expression, resulting in a reversal of EMT processes and a weakening of SW480 cell invasion and migration. In contrast, VPA significantly enhances cancer cell migration and invasion *in vitro*, and it can activate EMT in CRC cell lines, resulting in downregulation of epithelial markers: E-cadherin and ZO-1, and upregulation of mesenchymal markers: N-cadherin and fibronectin. In addition, VPA can significantly promote Snail expression through the Akt/GSK-3β signaling pathway.

### 5.2 *P. gingivalis* upregulates UCHL3 to promote CRC progression

UCHL3 (ubiquitin carboxy-terminal hydrolase L3) is an important transcription factor that promotes progression of colon cancer (Li et al., 2020). UCHL3 is positively associated with *P. gingivalis* infection in colon cancer; treatment of HCT116 cells or NCM460 cells with *P. gingivalis* significantly increased UCHL3 levels in a dose-dependent manner (Lu et al., 2023). The oncogenic mechanisms may be as follows (as shown in Figure 4): First, UCHL3 promotes EMT through the upregulation of Snail, Slug and ZEB1: deletion of UCHL3 leads to enhanced expression of E-cadherin, accompanied by decreased levels of vimentin and N-cadherin, as well as the transcription factors Slug, Snail and ZEB1. However, overexpression has the opposite effect. Second, UCHL3 activates the PI3K/AKT/mTOR pathway: UCHL3 knockdown results in a significant decrease in p-AKT levels. UCHL3 may induce the upregulation of sex-determining region Y-box12 (SOX12, which promotes certain tumors) by the PI3K/AKT/mTOR pathway. SOX12 can promote the synthesis of asparagine by upregulating glutaminase (GLS), glutamic-oxaloacetic transaminase 2 (GOT2), and asparagine synthetase (ASNS), which increases the progression of CRC (Du et al., 2019). Third, some experiments have concluded through mass spectrometry and protein blotting that UCHL3 may interact with GNG12 (G protein subunit gamma 12, a special G protein-coupled receptor that can participate in cancer immunity (Alausa et al., 2022). GNG12 promotes tumor progression by up-regulating PD-L1 through activation of the NF-κB pathway.

### 5.3 Activation of *RAS-RAF*-MEK-ERK signaling pathway by *P. gingivalis* promotes CRC cell proliferation

*RAS*, a frequently mutated oncogene, can activate the RAF kinase family (*ARAF*, BRAF, and CRAF, with BRAF itself being an oncogene), leading to increased phosphorylation levels that subsequently activate MEK1/2. The activated p-MEK1/2 then enhances ERK1/2 phosphorylation, which in turn increases the expression of the transcription factor activating protein 1 (AP-1), composed of proto-oncogenes Fos and Jun, regulating various cellular processes (Kim and Choi, 2015). *P. gingivalis* stimulates the *RAS-RAF*-MEK-ERK signaling pathway within 24 h, with gingival protease serving as the primary stimulant. Phosphorylation of key pathway components MEK1/2 and ERK1/2 is upregulated within 3–6 h (as shown in Figure 4). These findings suggest that *P. gingivalis* may promote the proliferation of CRC cells through this signaling pathway (Mu et al., 2020).

### 5.4 Activation of NLRP3 by *P. gingivalis* promotes CRC progression

At present, some studies have suggested that *P. gingivalis* promotes CRC by activating NLRP3 inflammasome. The mechanism involves the binding of LPS to Toll-like receptor 4 (TLR4), which subsequently activates NF-κB, leading to upregulation of NLRP3 expression (as shown in Figure 4) (Wang et al., 2021). The NLRP3 inflammasome is a multiprotein compound that induces three caspase-1-mediated reactions: IL-1β, IL-18, and pyroptosis. IL-1β, as a marker of NLRP3 inflammasome activation, is positively correlated with MMP9 and negatively correlated with E-cadherin, suggesting that IL-1β is involved in EMT and promotes the development of cancer (Marandi et al., 2021). IL-18 downregulates the soluble IL-22 receptor, known as IL-22 binding protein (IL-22BP). IL-22BP regulates the bioavailability of IL-22, which can inhibit early intestinal damage and promote tumor development over time (Sharma and Kanneganti, 2023).

In summary, *P. gingivalis* promotes colorectal tumor development through butyrate secretion, which may be associated with HDAC-mediated reduction of histone acetylation and induction of EMT-like phenotype. UCHL3 facilitates EMT by upregulating Snail, Slug, and ZEB1; it also activates the PI3K/AKT/mTOR pathway. Furthermore, UCHL3 interacts with and stabilizes GNG12, activating the NF-κB pathway that leads to PD-L1 upregulation and promotes tumor development. *P. gingivalis* gingipains activate the *RAS-RAF*-MEK-ERK signaling pathway and enhance AP1 expression, thereby stimulating CRC cell proliferation. Additionally, LPS binding to TLR4 activates NF-κB, which upregulates NLRP3 expression and triggers caspase-1-mediated production of IL-1β, IL-18, and pyroptosis, consequently accelerating tumor progression (as shown in Figure 4).

## 6 Pancreatic cancer

Pancreatic was once thought to be a sterile organ, but with increasing research, it has been found that the pancreas is not sterile and human pancreatic cancer (PC) tissues are rich in intra-tumoral microbiota (Wong-Rolle et al., 2021). The pathogenesis of PC may involve microbiota-induced local inflammation, which impair the host anti-tumor immune surveillance and change the tumor microenvironment (Pourali et al., 2024). Studies have found that the abundance of *P. gingivalis* in the control group without pancreatic cancer is significantly lower than that in PC patients (Ma et al., 2023). *P. gingivalis* can also be found in human pancreatic intraepithelial neoplasia (PanIN) lesions. And repeated administration of *P. gingivalis* to wild-type mice can cause pancreatic acinar duct metaplasia (ADM), thus *P. gingivalis* may accelerate the progression of PanIN to pancreatic ductal adenocarcinoma (PDAC) (Saba et al., 2024). We will further describe how *P. gingivalis* promotes the progression of PC next:

### 6.1 *P. gingivalis* promotes PC by promoting TME formation and the elevation of NE

**FIGURE 5:**
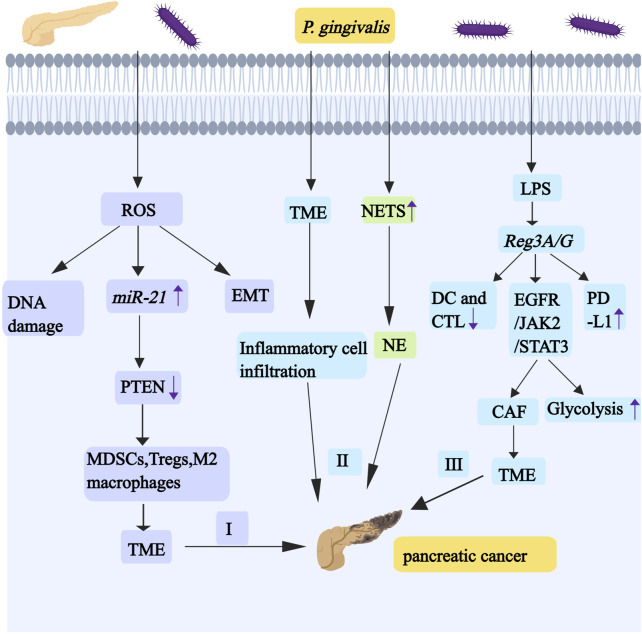
*P. gingivali*s promotes the development and progression of pancreatic cancer by regulating *miR-21*/PTEN axis, TME,NE and *Reg3A/G*. I, Infections with oral-intestinal pathogens (such as *P. gingivalis*) have the effect of chronically producing ROS, and ROS activates the *miR-21*/PTEN axis. The downregulation of PTEN causes cancer cells to be in a TME regulated by MDSCs, Tregs, and M2 macrophages. The production of ROS promotes EMT and causes DNA damage. II, *P. gingivalis* induces pro-inflammatory TME formation and the elevation of NE. III, *P. gingivalis* can upregulate the expression of *Reg3A/G* through its LPS, and *Reg3A/G* activates the EGFR/JAK2/STAT3 signaling pathway. STAT3 signaling can affect aerobic glycolysis. IL-1 enhances the heterogeneity and inflammation of CAF by participating in the JAK/STAT pathway, which contributes to the formation of the TME. *Reg3G* can inhibit the maturation of DCs and CTL activity. *Reg3G* also promotes the expression of PD-L1. Abbreviations: TME, tumorous microenvironment; MDSCs, myelogenous suppressor cell; Tregs, regulatory T cells; CAF, cancer-associated fibroblasts; DCs, dendritic cells; CTL, cytotoxic T lymphocyte; PD-L1, programmed death ligand 1.

*P. gingivalis* contributes to the formation of a pro-inflammatory tumor microenvironment (TME) and the elevation of neutrophil elastase (NE), thereby facilitating the progression of PC (as shown in Figure 5). Evidence indicates that *P. gingivalis*-treated tumors exhibit more pronounced necrosis and greater infiltration of inflammatory immune cells compared to untreated tumors (Tan et al., 2022). Immunohistochemical (IHC) analysis of myeloperoxidase (MPO) confirmed the presence of neutrophils, with cytopathic evaluations further supporting these findings. Additionally, RNA-seq reveals malignancy-associated TME signatures. Neutrophils, as the first line of defense against exogenous pathogens, engage in three primary mechanisms for microbial elimination: phagocytosis, degranulation, and the formation of neutrophil extracellular traps (NETs) (Sochalska and Potempa, 2017); The main component of NETs contains NE, which can promote cancer progression (Tan et al., 2022).

### 6.2 *P. gingivalis* activates the miR-21/PTEN axis to promote PC progression

Oral-intestinal pathogens may promote the escape of pancreatic cancer from host immune surveillance by activating the *miR-21*/PTEN axis—where *miR-21* serves as an oncogene in human cancers—and enhancing the presence of immunosuppressive cells (as shown in Figure 5). Infections with oral-intestinal pathogens have the effect of chronically producing ROS or stimulating inflammatory cytokines, including IL-1β, IL-6, and IL-8. IL-1β induces ROS and nitric oxide production through PI3K/Akt signaling. IL-6 drives EMT-mediated metastasis via cancer cell-derived ROS. ROS frequently cause DNA damage (Li R. et al., 2022). ROS can activate the expression and function of *miR-21* (Zhang Y. et al., 2020). *miR-21* downregulates PTEN, thereby promoting PC metastasis (Zhang et al., 2019). These pancreatic cancer cells are usually in immunosuppressive tumor microenvironment, which is regulated by regulatory T cells (Tregs), myeloid-derived suppressor cells (MDSCs) and M2 macrophages (Vidotto et al., 2020).

### 6.3 *P. gingivalis* upregulates *Reg3A/G* expression to promote PC development

*Reg3A/G* (*Reg3G*, a mouse homologue of human *Reg3A*) is a gene associated with pancreatic cancer (especially pancreatic ductal adenocarcinoma PDAC), and *P. gingivalis* can upregulate the expression of *Reg3A/G* through its LPS, suggesting that it may be an important target for the prevention of periodontal disease-associated pancreatic cancers (as shown in Figure 5) (Hiraki et al., 2020). *Reg3G* can inhibit the maturation of dendritic cells (DCs) and the anti-tumor effects of T cells, with the most pronounced inhibition of cytotoxic T lymphocyte (CTL) activity, and it also activates the EGFR/JAK2/STAT3 signaling pathway in tumors (Liu et al., 2017). AG490 is an EGF receptor (EGFR) inhibitor. When it treats PDAC cells, it can inhibit STAT3 and lead to reduced expression of cell cycle proteins, indicating that it has a great influence in the regulation of cell cycle and proliferation (Al-Hetty et al., 2023). When STAT3 is overexpressed ectopic, cell glucose consumption increases, which proves that STAT3 signaling can affect aerobic glycolysis. Cancer cells can promote the increase of glycolysis through Warburg effect, so as to better survive (Yucel et al., 2022). IL-1 enhances the heterogeneity and inflammation of cancer-associated fibroblasts (CAF) by participating in the JAK/STAT pathway, which contributes to the formation of the TME (as shown in Figure 5) (Biffi et al., 2019). *Reg3G* promotes the expression of programmed death ligand 1 (PD-L1), and binding of PD-L1 to its receptor programmed cell death 1 (PD-1) induces T cell inactivation and immune escape (as shown in Figure 5).

*P. gingivalis* promotes PC progression by inducing a proinflammatory TME and elevating NET. Specifically, this pathogen facilitates pancreatic cancer immune evasion both by activating the *miR-21*/PTEN axis and recruiting immunosuppressive cell populations, including MDSC, regulatory Treg, and M2 macrophages. PTEN downregulation creates an immunosuppressive TME dominated by these cell types. Moreover, *Reg3A/G* proteins not only impair DC maturation and T cell-mediated antitumor responses, but also activate the EGFR/JAK2/STAT3 signaling pathway, thereby dysregulating cell cycle progression and enhancing glycolysis. These mechanisms collectively induce PD-L1 upregulation and T cell dysfunction; consequently, they promote immune evasion and accelerate PC progression (as shown in Figure 5).

## 7 Rheumatoid arthritis

Rheumatoid arthritis (RA) is an autoimmune disease characterized by painful, swollen joints that cause damage to both the joints and the organs outside the joints, including the lungs, heart, digestive system, kidneys, eyes, skin, and nervous system (Gravallese and Firestein, 2023). Studies have demonstrated the effect of *P. gingivalis* on rheumatoid arthritis (Li Y. et al., 2022), and its DNA has been experimentally detected in synovial tissues. In RA patients, the concentration of anti-*P. gingivalis* antibodies is related to the expression of anti-citrulline protein antibodies (ACPA). Wegner tested 11 kinds of oral bacteria such as *P. gingivalis*, *Fusobacterium nucleatum, Aggregatibacter actinomycetemcomitans, Prevotella intermedia, Prevotella orali*. Wegner found that *P. gingivalis* was the only microorganism capable of producing endogenously guanosinated fibrinogen and enolase (Wegner et al., 2010). Among them, *Aggregatibacter actinomycetemcomitans* can create favorable colonization conditions for other organisms; it can suppress the host immune system and damage periodontal tissue, and the cytolethal distending toxin plays an important role in this process (Kim et al., 2023). *Prevotella intermedia* is associated with periodontal disease as well as other systemic diseases, including cystic fibrosis, chronic bronchitis, and atherosclerosis (Choe et al., 2019). The main virulence factors of *Prevotella intermedia* include LPS and cysteine protease, which have proinflammatory effects and promote immune escape (Zhang S. et al., 2024). Autoantibodies to the citrullinated forms of these antigens are highly specific for RA, and they play a causative role in the progression of the disease (Mikuls et al., 2012). Studies have suggested that this association may be based on the ability of *P. gingivalis* to express peptidyl deiminase (PAD), an enzyme responsible for citrullination of arginine residues post-translation, which can expose individuals to citrullinated antigens and easily lead to ACPA in the appropriate immunogenetic background, possibly contributing to the pathogenesis of RA synovitis (Hitchon et al., 2010). Smoking stimulates citrullination of the peptides PAD2 and PAD4, which is also an environmental risk factor for RA. These two findings suggest that positive anti-citrulline protein antibodies play a role in RA development (Krutyhołowa et al., 2022). Some scholars have constructed an animal model of MHC-type C3H mice with ACPA-positive RA that combines *P. gingivalis* infection and collagen immunity. Compared with the control group, the expression of ACPA and citrulline protein in this model was elevated, and there are significant changes in the subpopulations of immune cells, which may be a key factor in increasing bone destruction, inflammation, and pain (Yang et al., 2024). *P. gingivalis* induces the activation of M1 macrophages, which produce a variety of inflammatory cytokines that promote the progression of RA, such as TNF-α, IL-1β, IL-12. Overnutrition of RA macrophages leads to excessive glucose uptake, which in turn produces high levels of ATP and mitochondrial ROS, and high levels of ROS are involved in the destruction of joints and cartilage in RA. LPS can reduce the levels of oxidative phosphorylation and increases the levels of glycolysis in macrophages, which leads to the accumulation of intermediate metabolites of the tricarboxylic acid cycle such as succinic acid (Yang X. et al., 2020). Intracellular succinic acid induces angiogenesis via HIF-1α, whereas extracellular succinic acid acts on the activation of G protein-coupled receptor 91 (GPR91), which together disrupt energy metabolism and exacerbate inflammation and angiogenesis in the synovial membrane of arthritic joints (Li et al., 2018). Metabolism in M1 macrophages promotes the production of lactic acid and succinic acid, which acidify the extracellular space, leading to the formation of a low-glucose and high lactic acid microenvironment, which is typical of the microenvironment in RA (Lin et al., 2022).

## 8 Alzheimer's disease

Alzheimer’s disease (AD) is a disease associated with progressive neurodegeneration that is characterized by intracellular amyloid beta protein (Aβ) plaques in the brain and neurofibrillary tangles (NFT). Diagnosis currently relies on positron emission tomography (PET) with tracer molecules and analysis of proteins in the cerebrospinal fluid (CSF) (Khan et al., 2020). Studies have proved that Alzheimer’s disease is strongly associated with dysbiosis (Zhang Y. et al., 2024). Various *P. gingivalis* biomolecules have been identified in the cortical gray matter, basal forebrain and hypothalamus regions of the human AD brain.

**FIGURE 6:**
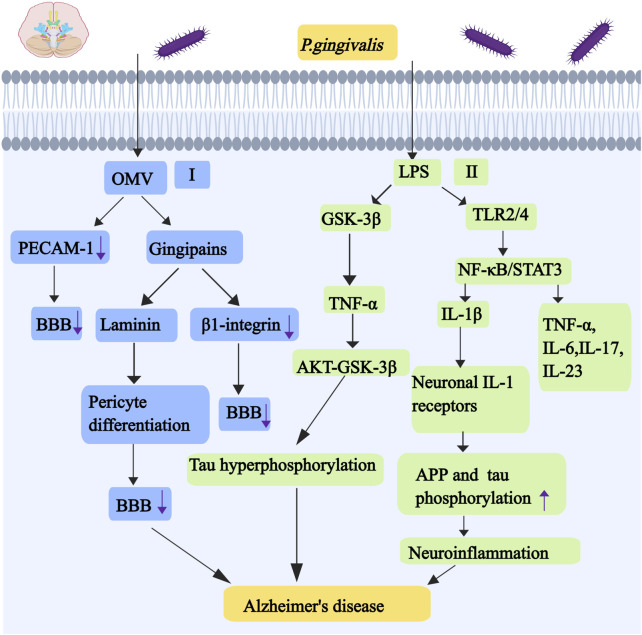
Mechanisms associated with alzheimer’s disease caused by *P. gingivalis*. I, *P. gingivalis* OMVs can cleave endothelial adhesion protein blood plates/endothelial cell adhesion molecule-1 (PECAM-1). Gingipains have a strong affinity for astrocyte laminin, and astrocyte laminin can regulate pericyte differentiation to affect the integrity of the BBB. Gingipains also degrade β1-integrin,and the deficiency of β1-integrin in endothelial cells resulted in an imperfect BBB and significant cerebral hemorrhage. II, LPS of *P. gingivalis* activates GSK-3β, leading to increased expression of TNF-α. TNF-α can trigger AKT-GSK-3β-mediated tau hyperphosphorylation in neurons. The activation of NF-κB pathway by LPS of *P. gingivalis* promotes the production of cathepsin B, cathepsin B leads to the production of IL-1β. IL-1β acts on neuronal IL-1 receptors and promotes the expression and processing of APP and tau phosphorylation in neurons that can cause chronic activation of neuroinflammation. LPS of *P. gingivalis* can activate NF- κ B and STAT3 pathways by binding to TLR2 and TLR4, which can increase the expression and secretion of proinflammatory cytokines, including TNF- α, IL-1 β, IL-6,IL-17 and IL-23. Abbreviations: PECAM-1, endothelial cell adhesion molecule-1; BBB, blood-brain barrier; APP, amyloid precursor protein.

Aspiration of oral bacteria aggravates lung disease and gastrointestinal microbiota-gut-brain axis disorder. It induces chronic systemic inflammation, which manifests itself in the brain as an increased neuroinflammatory load (Xue et al., 2020). At the same time, the epithelial damage in the inflamed periodontal pocket makes it easier for periodontal bacteria to invade the adjacent primary afferent nerves and blood vessels. Periodontal bacteria that escape from the mouth go directly into the brain along these nerves and blood vessels, causing brain infections. This brain inflammation causes activated microglia and reactive astrocytes to produce inflammatory mediators that promote the production of Aβ. The accumulation of Aβ leads to hyperphosphorylation of Tau, which constitutes the histopathological signature of AD (Selkoe and Hardy, 2016). *P. gingivalis* OMVs can disrupt and penetrate the blood-brain barrier (BBB) by a number of mechanisms, and gingipains enriched on the surface of OMV can cleave a number of protein components of the barrier, including extracellular matrix proteins, connexins and integrins (Zhao et al., 2015). *P. gingivalis* OMVs can cleave endothelial adhesion protein blood plates/endothelial cell adhesion molecule-1 (PECAM-1) that is present at the BBB adhesion junction,and vascular permeability can be increased *in vitro* in a gingivalis-dependent manner (as shown in Figure 6) (Farrugia et al., 2020). Gingipains degrade β1-integrin. The deficiency of β1-integrin in endothelial cells resulted in an imperfect blood-brain barrier and significant cerebral hemorrhage in mice (Qiu et al., 2018). Gingipains have a strong affinity for several extracellular matrix proteins, such as astrocyte laminin, which can regulate pericyte differentiation to affect the integrity of the blood-brain barrier (as shown in Figure 6) (Yao et al., 2014). LPS of *P. gingivalis* can activate NF- κB and STAT3 pathways by binding to TLR2 and TLR4 on microglia, which can increase the expression and secretion of proinflammatory cytokines, including TNF- α, IL-1 β, IL-6, IL-17 and IL-23 (Qiu et al., 2021). Specifically, the activation of NF-κB pathway by LPS of *P. gingivalis* promotes the production of cathepsin B in microglial cells, which leads to the production of IL-1β in microglia. IL-1 β acts on neuronal IL-1 receptors and promotes the expression and processing of amyloid precursor protein (APP) and tau phosphorylation in neurons that can cause chronic activation of neuroinflammation (as shown in Figure 6) (Liu et al., 2024). LPS of *P. gingivalis* activates GSK-3β in microglia, leading to increased expression of TNF-α, which can trigger AKT-GSK-3β-mediated tau hyperphosphorylation in neurons (as shown in Figure 6) (Jiang et al., 2021). Gingipains may play a key role in this process, and inhibition with gingipains can reduce the bacterial load of *P. gingivalis* brain infection, which can reduce neuroinflammation and save neurons in the hippocampus (Dominy et al., 2019). Although all strains can invade the brain, only the most virulent *P. gingivalis* strains K1 and K2 effectively induce proinflammatory cytokine production, astrogliosis, Aβ secretion, Tau hyperphosphorylation, and cognitive decline under short exposure to infection (Díaz-Zúñiga et al., 2020). Regarding the role of gingipains in AD, gingipain inhibitors (COR388, Atuzaginstat) have been developed. This small-molecule inhibitor specifically targets gingipains, reducing *P. gingivalis* brain infection, decreasing Aβ deposition, and improving cognitive function in animal models. In clinical trials, COR388 treatment slowed cognitive decline by 57% in Pg-positive AD patients (measured by ADAS-Cog11 scores). The next-generation inhibitor, LHP588, exhibits superior blood-brain barrier permeability. Currently undergoing Phase 2 trials, LHP588 demonstrates significant therapeutic promise for AD treatment.

## 9 Cardiovascular disease

Studies have shown that oral flora dysbiosis and the enrichment of certain specific pathogenic bacteria are strongly associated with heart-related diseases (Ghanem et al., 2024). The pathogenesis of atherosclerosis by *P. gingivalis* may start from the stage of promoting the pathological changes of lipid metabolism, which induces the oxidation of high-density lipoproteins and compromises the atherosclerotic protective function of these lipoproteins (Kim et al., 2018). *P. gingivalis* and its gingipains promote the production of ROS and consume antioxidants, such as Rgp and Kgp, which can cause lipid peroxidation (Lönn et al., 2018). Entry of *P. gingivalis* into vascular endothelial cells was positively correlated with bacterial load as well as with some virulence proteins such as gingipains, fimbria, and hemagglutinin A. The Kgp significantly causes endothelial homeostasis imbalance by reducing plasminogen activator inhibitor-1 levels in endothelial cells, ultimately leading to permeability and dysfunction of the vascular endothelial barrier (Song et al., 2022). *P. gingivalis* can cause cells to lose the ability of self-repair by inhibiting endothelial cells proliferation, promoting endothelial mesenchymal transition and endothelial cell apoptosis. *P. gingivalis* promotes the expression of macrophage migration inhibitory factor (MIF) in endothelial cells, which stimulates the secretion of proinflammatory cytokines by macrophages, prolonging macrophage survival and exacerbating inflammatory responses (Xu et al., 2018). Macrophage migration inhibitors enhance the formation of foam cells (Chen et al., 2017). Gingipains have effects on the proliferation and phenotypic transformation of smooth muscle cells (SMC) in rats (Cao et al., 2015). The fragmentation of vascular IV collagen can be caused by matrix metallopeptidase 9 produced by macrophages that are induced by *P. gingivalis*. *P. gingivalis* infection not only leads to platelet activation and aggregation, but also increases the expression of P-selectin and the binding of fibrinogen to platelets, which may promote thrombosis, increasing the risk of thrombus formation (Czerniuk et al., 2022).

## 10 Respiratory diseases

At present, studies have suggested that the abundance of *P. gingivalis* in patients with chronic obstructive pulmonary disease (COPD) was significantly higher than that in the non-COPD group (Wu et al., 2017). *P. gingivalis* in the tracheal aspirates of patients with AECOPD was highly homologous to the strains present in dental plaque, and the number of *P. gingivalis* was higher than that in the mouth (Tan et al., 2014). The above studies have shown that COPD was related to *P. gingivalis*. Compared with the normal control group, the relative content of *P. gingivalis* in patients with COPD was significantly negatively correlated with FEV1% (Tan et al., 2019). Compared with neighboring lung tissues, the positivity rate of *P. gingivalis* staining slices in cancer tissues of small-cell lung cancer, pulmonary adenocarcinoma, and pulmonary squamous cell carcinoma was higher, and the survival rate and median survival time of lung cancer patients were also significantly reduced due to bacterial infection with *P. gingivalis* (Liu Y. et al., 2021). Some studies have found a positive correlation between the risk of lung cancer and the level of IgG antibodies to *P. gingivalis* (Ampomah et al., 2023). Clinical data further demonstrate that patients testing positive for *P. gingivalis* infection demonstrate significantly reduced overall survival, with the most statistically significant difference observed in lung squamous cell carcinoma (LSCC) patients. *P. gingivalis* is a potent inflammatory stimulant in human bronchial and pharyngeal epithelial cells, stimulating the production of TLR2-mediated cytokines IL-6 and IL-8, which may lead to episodes of aspiration pneumonia (Watanabe et al., 2020). Intratracheal inoculation of *P. gingivalis* W83 in female BALB/c mice significantly increases TNF-α, IL-6, monocyte chemotactic protein-1 (MCP-1), and CRP levels within 24 h compared to gingipain-deficient mutants (Benedyk et al., 2016). TNF-α is an effective activator of NF-κB that promotes neutrophil inflammation and activates macrophages, which may be implicated in the development of lung cancer and COPD (Benoot et al., 2021). MCP-1 recruits and activates inflammatory cells in the airways, including T lymphocytes and B lymphocytes and monocytes. Gingipains may play a role in the production of these proinflammatory cytokines. Mice inoculated with a strain of *P. gingivalis* that knocked out gingivalis-related genes showed only brief and mild pneumonia with no significant change in pro-inflammatory cytokines, whereas mice inoculated with wild-type *P. gingivalis* showed symptoms of respiratory failure (Shi et al., 2023).

## 11 Other diseases

*P. gingivalis* not only promotes the occurrence and development of the above diseases, but also is strongly associated with diabetes, depression, metabolic dysfunction-associated fatty liver disease (MAFLD), adverse pregnancy outcomes and other diseases.

Diabetes is a metabolic disease characterized by high blood sugar (Harreiter and Roden, 2023). Studies have found that treatment of periodontal disease leads to improved metabolic control in diabetic patients (Vergnes, 2010). Lipopolysaccharide of gram-negative bacteria can lead to insulin resistance (Cani et al., 2007). Periodontal dysbiosis may first trigger regional metabolic inflammation and then systemic metabolic inflammation, promoting insulin resistance and type 2 diabetes mellitus (T2DM) (Blasco-Baque et al., 2017). *P. gingivalis* OMVs attenuate insulin-induced Akt/GSK-3β signaling in a gingipain-dependent manner, inducing changes in glucose metabolism in the liver and contributing to the progression of diabetes (Seyama et al., 2020). Clinical evidence suggests that T2DM and periodontitis show higher susceptibility to the highly virulent FimA genotype II strain of *P. gingivalis* compared to periodontitis patients without systemic diseases. The use of antimicrobial mouthwash in T2DM patients has been shown to significantly reduce oral colonization of *P. gingivalis* and other periodontal pathogens, with associated reductions in glycated hemoglobin (HbA1c) levels observed in some patients.

Metabolic dysfunction-associated fatty liver disease is classified by histologic features into nonalcoholic fatty liver (NAFL) and nonalcoholic steatohepatitis (NASH) (Guo et al., 2022). Epidemiologic investigations suggest that a history of periodontitis may be an independent risk factor for MAFLD (Akinkugbe et al., 2017). *P. gingivalis* infection leads to disruption of the oral mucosal barrier, causing bacteremia and endotoxemia that may accelerate the progression of MAFLD (Wang T. et al., 2022). *P. gingivalis* may contribute to the progression of NASH by activating the LPS-TLR2 pathway and inflammatory vesicles (Furusho et al., 2013). *P. gingivalis* LPS may promote intracellular lipid accumulation and inflammatory responses in HepG2 cells (a type of liver tumor cell line) by activation of the NF-κB and JNK signaling pathways (Ding et al., 2019).

Severe depression is one of the most common personal and public health conditions in the world that can debilitate people (Monroe and Harkness, 2022). Studies have found that periodontitis is an independent risk factor for depression (Hsu et al., 2015). The hypothesis of deficiency of neurotrophic factors in the pathogenic mechanism of depression refers to the reduction of neurotrophic factors that prevents the brain from adapting to environmental stimuli, leading to the onset of depression (Duman et al., 1997). LPS from *P. gingivalis* activates astrocytes and downregulates p75NTR via TLR4, which inhibits the maturation of brain-derived neurotrophic factor (BDNF) and ultimately promotes depression (Wang et al., 2019).

Adverse pregnancy outcomes generally include ectopic pregnancy, spontaneous abortion, stillbirth, premature delivery,low birth weight fetuses, gestational diabetes mellitus (GDM) and hypertensive disorders of pregnancy (Farland et al., 2019). Studies have found that periodontal disease is associated with adverse pregnancy outcomes (Figuero et al., 2020). LPS of *P. gingivalis* has an effect on the growth of placenta and fetus (Kunnen et al., 2014). *P. gingivalis* may induce the production of antibodies that cross-react with beta2-glycoprotein I (beta2GPI), the target antigen of autoimmune anticardiolipin antibodies (aCL), which cause abortion in mice (Schenkein et al., 2013). Dentists should provide safe periodontal disease treatment for pregnant women to reduce some adverse effects (Bobetsis et al., 2020).

## 12 Discussion

*P. gingivalis* produces many virulence factors such as OMVs, LPS, gingipains, and fimbria, which allow *P. gingivalis* to better invade the organism and lead to the development of a series of diseases in the host (as shown in Table 1). Among the diseases mentioned in this paper, the diseases caused by LPS activating NF-κB are periodontal disease (Ding et al., 2013), esophageal cancer (Meng et al., 2019), colorectal cancer (Wang et al., 2021), Alzheimer’s disease (Qiu et al., 2021), cardiovascular disease (Lei et al., 2011) and MAFLD (Ding et al., 2019). The diseases of GSK-3β activated by LPS are oral cancer (Lee et al., 2017), esophageal cancer (Liu et al., 2023) and Alzheimer’s disease (Jiang et al., 2021). STAT3 activation by LPS is associated with esophageal cancer (Gao et al., 2017) and Alzheimer’s disease (Qiu et al., 2021). *P. gingivalis* LPS activates astrocytes, which inhibits brain-derived neurotrophic factor (BDNF) maturation and promotes depression (Wang et al., 2019). LPS can also affect the adverse pregnancy outcomes (Kunnen et al., 2014). The pathway activated by *P. gingivalis* LPS mainly promotes the production of inflammatory cells, and fimbriae can help LPS bind to TLR2. *P. gingivalis* Mfa1 may bind to TLR2, which can induce human bronchial epithelial cells to produce IL-6 and IL-8 (Takahashi et al., 2022). The diseases caused by gingipains virulence factors are periodontal disease (Imamura, 2003; Cichońska et al., 2024), esophageal cancer (Malinowski et al., 2019), colorectal cancer (Mu et al., 2020), Alzheimer’s disease (Zhao et al., 2015), aspiration pneumonia (Benedyk et al., 2016) and RA (Ahmadi et al., 2023). Gingipains can cleave IgG in the Fc region and convert it into rheumatoid factor (RF) antigen. OMVs can promote the occurrence and development of AD (Zhao et al., 2015) and diabetes (Seyama et al., 2020). Other studies have suggested that it is related to RA, because 78 citrullinated proteins have been found in OMVs (Larsen et al., 2020). OMVs can also increase vascular permeability and promote cardiovascular disease, which may be related to the mechanism of proteolysis of endothelial cell adhesin molecules (such as PECAM-1) (Farrugia et al., 2020). *P. gingivalis* can promote the occurrence of oral cancer (Abdulkareem et al., 2018), colorectal cancer (Wawruszak et al., 2019) and pancreatic cancer (Li R. et al., 2022) by inducing EMT formation. From the above, we can find that the diseases promoted by *P. gingivalis* have a common pathway, but some of them are different. Reasons for this effect may be that many virulence factors of *P. gingivalis* play a strong or weak role and the occurrence of the diseases in various parts of the body.

**TABLE 1:** *P. gingivalis* virulence factors contribute to disease mechanisms.

Virulence factors	Mechanism	Related diseases
LPS	NF-κB	Periodontal disease (Ding et al., 2013), Esophageal cancer (Meng et al., 2019), Colorectal cancer (Wang et al., 2021), Alzheimer’s disease (Qiu et al., 2021), Cardiovascular disease (Lei et al., 2011) and MAFLD (Ding et al., 2019)
	GSK-3β	Oral cancer (Lee et al., 2017), Esophageal cancer (Liu et al., 2023) and Alzheimer’s disease (Jiang et al., 2021)
	STAT3	Esophageal cancer (Gao et al., 2017) and Alzheimer’s disease (Qiu et al., 2021)
	BDNF	Depression (Wang et al., 2019)
	hypertension	The adverse pregnancy outcomes (Kunnen et al., 2014)
Gingipains	MMP	Periodontal disease (Imamura, 2003; Cichońska et al., 2024), Esophageal cancer (Malinowski et al., 2019)
	*RAS-RAF*-MEK-ERK	Colorectal cancer (Mu et al., 2020)
	degradation of protein components of BBB	Alzheimer’s disease (Zhao et al., 2015)
	systemic inflammatory responses	Aspiration pneumonia (Benedyk et al., 2016)
	inflammatory reaction and RF	RA (Ahmadi et al., 2023)
OMVs	degradation of protein components of BBB	AD (Zhao et al., 2015)
	Akt/GSK-3β	Diabetes (Seyama et al., 2020)
	citrullinated proteins	RA (Larsen et al., 2020)
	PECAM-1	Cardiovascular disease (Farrugia et al., 2020)
*P. gingivalis*	EMT	Oral cancer (Abdulkareem et al., 2018), Colorectal cancer (Wawruszak et al., 2019) and Pancreatic cancer (Li et al., 2022a)

This review synthesizes extensive literature on *P. gingivalis* through critical evaluation and systematic summarization, deliberately excluding studies with unclear or unsubstantiated mechanisms to ensure the objectivity and scientific validity of the presented pathogenic mechanisms. Nevertheless, certain limitations must be acknowledged. Some disease-related research remains confined to *in vitro* experiments, resulting in a translational gap for clinical applications, while other mechanistic observations lack sufficient experimental data for comprehensive interpretation. Emerging therapeutic strategies against *P. gingivalis*-associated diseases warrant discussion. A novel detection method utilizing recombinase polymerase amplification combined with lateral flow (RPA-LF) technology has been successfully developed, demonstrating clinical potential for point-of-care diagnosis. The FimA genotype of *P. gingivalis*, particularly prevalent in diabetic populations, correlates with heightened virulence and may serve as a molecular biomarker for high-risk individuals. Probiotic interventions involving the gut commensal Akkermansia muciniphila have shown promise—this bacterium enhances oral mucosal immunity via the TLR-MYD88-NF-κB pathway, upregulates antimicrobial peptide expression, and inhibits *P. gingivalis* adhesion and invasion (Hu et al., 2025). Exploiting *P. gingivalis*’ heme-dependent growth, erythrocyte-mimicking nanovesicles loaded with gallium protoporphyrin can be selectively internalized by the bacterium. Subsequent blue light irradiation generates ROS to achieve targeted bactericidal effects while preserving commensal microbiota. The aforementioned gingipain inhibitor COR388 (Atuzaginstat) has exhibited potential in slowing cognitive decline in AD patients. Integrated multi-omics approaches, incorporating genomic, proteomic, and metabolomic data, could refine personalized therapeutic strategies. Furthermore, interdisciplinary convergence of microbiology, immunology, and materials science is poised to advance precision-targeted interventions against *P. gingivalis*.

Through investigating the relationship between *P. gingivalis* and these diseases, we can gain deeper insights into its pathogenic mechanisms in disease development. This article primarily examines the pathological mechanisms underlying P. gingivalis-associated diseases. However, specific mechanisms of *P. gingivali*s virulence factors for some diseases are not clear at present, which require further research and discussion. Our research has demonstrated that reducing *P. gingivalis* colonization significantly benefits oral health maintenance, particularly in preventing and controlling periodontal disease progression. How to eradicate *P. gingivalis* and the relationships between *P. gingivalis* and other oral pathogens and intestinal microbiota are topics that we need to study next. The multi omics approach combining metagenomics, transcriptomics, and metabolomics may become an important breakthrough for future research.
